# Effect of galectin‐1 on prognosis and responsiveness of immune checkpoint plus tyrosine kinase inhibition in renal cell carcinoma

**DOI:** 10.1002/cam4.7113

**Published:** 2024-03-28

**Authors:** Jiajun Wang, Sihong Zhang, Ying Wang, Yanjun Zhu, Xianglai Xu, Jianming Guo

**Affiliations:** ^1^ Department of Urology, Zhongshan Hospital Fudan University Shanghai China; ^2^ Department of Critical Care Medicine, Zhongshan Hospital Fudan University Shanghai China

**Keywords:** galectin‐1, immune checkpoint inhibitor, renal cell carcinoma, T cell exhaustion, tyrosine kinase inhibitor

## Abstract

**Background:**

In renal cell carcinoma (RCC), no clinically available biomarker has been utilized for checkpoint inhibitor immunotherapy (IO) + tyrosine kinase inhibitor (TKI) combinations. Galectin‐1 overexpression is found in tumors, with potential immune‐regulating roles.

**Methods:**

RNA‐sequencing was performed in two cohorts of RCC treated with IO/TKI combination therapy (ZS‐MRCC, JAVELIN‐101). Immunohistochemistry and flow cytometry were performed to investigate immune cell infiltration and function in the tumor microenvironment of RCC. The RECIST criteria were used to define response and progression‐free survival (PFS).

**Results:**

Galectin‐1 expression was elevated in RCC with higher stage (*p* < 0.001) and grade (*p* < 0.001). Galectin‐1 expression was also elevated in non‐responders of IO/TKI therapy (*p* = 0.047). High galectin‐1 was related with shorter PFS in both ZS‐MRCC cohort (*p* = 0.036) and JAVELIN‐101 cohort (*p* = 0.005). Multivariate Cox analysis defined galectin‐1 as an independent factor for PFS (HR 2.505; 95% CI 1.116–5.622; *p* = 0.026). In the tumor microenvironment, high galectin‐1 was related with decreased GZMB+CD8+ T cells (Speraman's *ρ* = −0.31, *p* = 0.05), and increased PD1 + CD8+ T cells (Speraman's *ρ* = 0.40, *p* = 0.01). Besides, elevated number of regulatory T cells (*p* = 0.039) and fibroblasts (*p* = 0.011) was also found in high galectin‐1 tumors. Finally, a random‐forest score (RFscore) was built for predicting IO/TKI benefit. IO/TKI therapy showed benefit only in low‐RFscore patients (HR 0.489, 95% CI 0.358–0.669, *p* < 0.001), rather than high‐RFscore patients (HR 0.875, 95% CI 0.658–1.163, *p* = 0.357).

**Conclusions:**

High galectin‐1 indicated therapeutic resistance and shorter PFS of IO/TKI therapy. High galectin‐1 also indicated CD8+ T cell dysfunction. High galectin‐1 could be applied for patient selection of IO/TKI therapy in RCC.

## Graphical abstract

We found that high galectin‐1 expression indicated therapeutic resistance and shorter PFS of IO/TKI therapy in mRCC. High galectin‐1 expression was also related with CD8+ T cell dysfunction and immune evasion in RCC. The integrated RFscore of galectin‐1 expression and other immunologic factors may guide treatment selection between IO/TKI versus TKI monotherapy in mRCC.

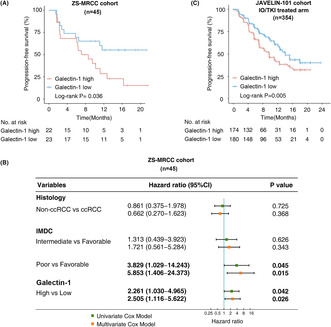

## 1 INTRODUCTION

Renal cell carcinoma (RCC) is one of the most commonly diagnosed solid tumors.^1^, ^2^ Approximately 25% of RCC patients are diagnosed with advanced, unresectable, or metastatic illness, which causes cancer‐related death.^1^, ^2^ In 1992, high‐dose interleukin‐2 (IL‐2) was approved as the first immunotherapy (IO) strategy for patients with metastatic RCC (mRCC).^3^ However, this regimen only had a complete response (CR) rate of 5%–9%.^3^ Immune checkpoint inhibitors (ICIs) targeting programmed cell death protein 1 (PD‐1), programmed death‐ligand 1 (PD‐L1), and cytotoxic T‐lymphocyte‐associated protein 4 (CTLA‐4) have been used to treat a variety of cancers. For mRCC, the combination of IO and tyrosine kinase inhibitor (TKI) has been applied as the first‐line recommendation in recent years.^4^ However, despite the encouraging clinical outcome, the combination of IO/TKI is only effective in a subgroup of patients, and there is no reliable companion diagnostic biomarker available.^5^, ^6^, ^7^

Galectin‐1, encoded by LGALS1, is one of the members of the galectin family of β‐galactoside‐binding proteins.^8^ Galectin‐1 is an important immunosuppressive molecule across cancer types.^9^ Galectin‐1 released by tumor can attach to glycosylated receptors on immune cells, inhibiting immune cell activity in the tumor microenvironment.^10^ In animal models, tumor‐secreted galectin‐1 also promotes immune evasion by blocking T cell recruitment into the tumor.^11^ Furthermore, galectin‐1 treatment of activated T cells increased the release of Th2 cytokines and the expansion of regulatory T cells (Tregs).^12^

As a critical immunosuppressive molecule across cancer types, galectin‐1 may also contribute to responsiveness to IO monotherapy and IO‐based combos.^9^ An in silico analysis found that galectin‐1 expression could predict the responsiveness to anti‐PD1 therapy.^13^ Galectin‐1 reprograms the tumor epithelium by upregulating cell‐surface PD‐L1, leading to T cell exclusion.^11^ The findings suggested that galectin‐1 may have a role in determining IO response. Galectin‐1 expression correlated inversely with treatment response and survival in patients with head and neck cancer treated with ICIs.^11^ However, the prognostic and predictive effect of galectin‐1 in IO/TKI combinations in RCC has not yet been investigated.

In this study, we aimed to investigate the prognostic and predictive significance of galectin‐1 for IO/TKI combinations in RCC. The relationships between galectin‐1 and treatment response and survival were investigated in cohorts with metastatic RCC treated with IO/TKI. This study sheds light on how galectin‐1 promotes immune evasion and IO/TKI resistance in RCC.

## 2 MATERIALS AND METHODS

### 2.1 Study cohorts and data collection

The study included four independent cohorts: the ZS‐MRCC cohort, the JAVELIN‐101 cohort, the ZS‐HRRCC cohort, and the TCGA‐KIRC cohort. Detailed clinical and pathologic information of the cohorts has been described in our previous research.^14^ The ZS‐MRCC cohort included 45 metastatic RCC patients treated with IO/TKI in Zhongshan Hospital, Fudan University. Inclusion criteria, exclusion criteria and baseline characteristics of the ZS‐MRCC cohort have been described in our previous research.^14^ Response and progression were assessed according to the RECIST 1.1 criteria.^15^ For the ZS‐MRCC cohort, tissue collection and further tests were performed by our team. The JAVELIN‐101 cohort included 726 metastatic RCC patients, treated by IO/TKI (*n* = 354) or TKI monotherapy (*n* = 372), from the JAVELIN Renal 101 clinical trial.^5^ Inclusion criteria, exclusion criteria and patients' characteristics, survival, genomic and transcriptomic data were acquired from the previous studies of JAVELIN Renal 101 clinical trial.^5^, ^16^ For the JAVELIN‐101 cohort, tissue collection and further tests were performed in the previous study by Motzer et al.^16^ The ZS‐HRRCC cohort included 40 high‐risk localized RCC patients from Zhongshan Hospital, Fudan University, which has been described in our previous research.^14^ For the ZS‐HRRCC cohort, tissue collection and further tests were performed by our team. The TCGA‐KIRC cohort enrolled 530 patients of clear cell renal cell carcinoma as part of the Cancer Genome Atlas (TCGA) project.

The study has been approved by the Clinical Research Ethics Committee of Zhongshan Hospital, Fudan University (B2021‐119), and the Declaration of Helsinki was obeyed. Informed consent was obtained from each participate.

### 2.2 RNA‐sequencing and data processing

RNA‐sequencing and data processing procedures have been described in our previous research.^14^ Total RNA isolation was performed by using MagBeads Total RNA Extraction Kit (MAJORIVD). Total RNA purification was performed by using RNAClean XP Kit (Beckman Coulter) and RNase‐Free DNase Set (QIAGEN). Library construction and sequencing was performed by Shanghai Biotechnology Corp. (Shanghai, China), using VAHTS Universal V6 RNA‐sequencing Library Prep Kit for Illumina (Vazyme) and NovaSeq 6000 equipment (Illumina). Read count value and FPKM was utilized for RNA‐sequencing data standardization.

### 2.3 Hematoxylin & eosin staining and immunohistochemistry

Hematoxylin & eosin (H&E) staining and immunohistochemistry were performed on formalin‐fixed, paraffin‐embedded samples in a previously‐described cohort of highrisk localized RCC from our institution (ZS‐HRRCC).^17^ Tumor‐infiltrating lymphocytes (TILs) were assessed following standard procedures according to H&E.^18^ Immunohistochemistry was performed as described before.^19^ Primary antibodies are listed in Table S2. Digital images were scanned by PANNORAMIC® 250 Flash III DX system (3DHISTECH Ltd.) and browsed by CaseViewer application (3DHISTECH Ltd.). Immune cell infiltration was assessed by three independent investigators masked to patients' information, under six fields. The average values were calculated for further analysis.

### 2.4 Flow cytometry

Flow cytometry procedures were performed in the ZS‐HRRCC cohort as previously described.^17^ In brief, Peripheral blood samples were collected preoperatively, and white blood cells were extracted after adding RBC Lysis Buffer (Thermo Fisher Scientific). RCC tissues were collected after surgical resection, minced and then digested with collagenase IV (Sigma) and DNase I (Sigma), strained through a 70‐μm strainer, and then treated with RBC lysis buffer (Thermo Fisher Scientific). After Fc receptors blockade, staining with fluorescently labeled membrane marker antibodies was performed at 4°C for 30 min. For intracellular proteins, staining with fluorochrome‐labeled antibodies was performed after disposure by Intracellular Fixation & Permeabilization Buffer (Thermo Fisher Scientific). Flow cytometry data were collected by BD LSRFortessa™ X‐20 (BD Biosciences) and analyzed by Flowjo v10.0 (Tree Star). Detailed antibody information is provided in Table S2.

### 2.5 In silico approaches

In silico approaches were performed by R software (https://www.r‐project.org/). Survival analyses were performed by “survival” and “survminer” packages of R software. The forest plots were drawn using “forestplot” package of R software. Gene Set Enrichment Analysis (GSEA) was performed using MSigDB's hallmark gene sets^20^, ^21^ by “clusterProfiler” R package.^22^ The waterfall plot was drawn by “ComplexHeatmap” and “ggplot2” packages of R software. The random forest model was constructed by “randomForestSRC” and “ggRandomForests” packages of R software.

### 2.6 Random forest model construction

Random forest classification algorithm is an ensemble learning method for classification and regression.^23^ It is a nonparametric approach suitable for analyzing survival data and complex omics data.^24^, ^25^ In the study, tumor microenvironment parameters including LGALS1, PDCD1, GZMB, CD8A, GZMK, CTLA4, CD4, and CD274 expression were enrolled for the construction of the random forest score (RFscore). High/low expression was set by the median. For model construction, high expression was identified as 1, and low expression was identified as 0. The random forest model construction was performed by “randomForestSRC” and “ggRandomForests” packages of R software, by the following code: modRFSRC ≤ rfsrc (Surv (futime, fustat) ~ ., data = data1, ntree = 5000, nodesize = 15, block.size = 1, na.action = “na.omit”).

### 2.7 Statistical analysis

Categorical variables were analyzed by Chi‐square test, Fisher's exact analysis or Cochran–Mantel–Haenszel test. Continuous variables were analyzed between groups by Wilcoxon signed‐rank test or Kruskal–Wallis *H*‐test. Correlations were analyzed by Spearman's correlation analysis. For continuous variables, high‐ and low‐ groups were divided by median values. Survival analyses were performed by Kaplan–Meier analyses and Cox regression models. All Statistical analyses were performed by R software (https://www.r‐project.org/). *p*‐value < 0.05 was regarded as statistically significant.

## 3 RESULTS

### 3.1 Elevated galectin‐1 expression correlated with RCC progression and aggressiveness

**FIGURE 1:**
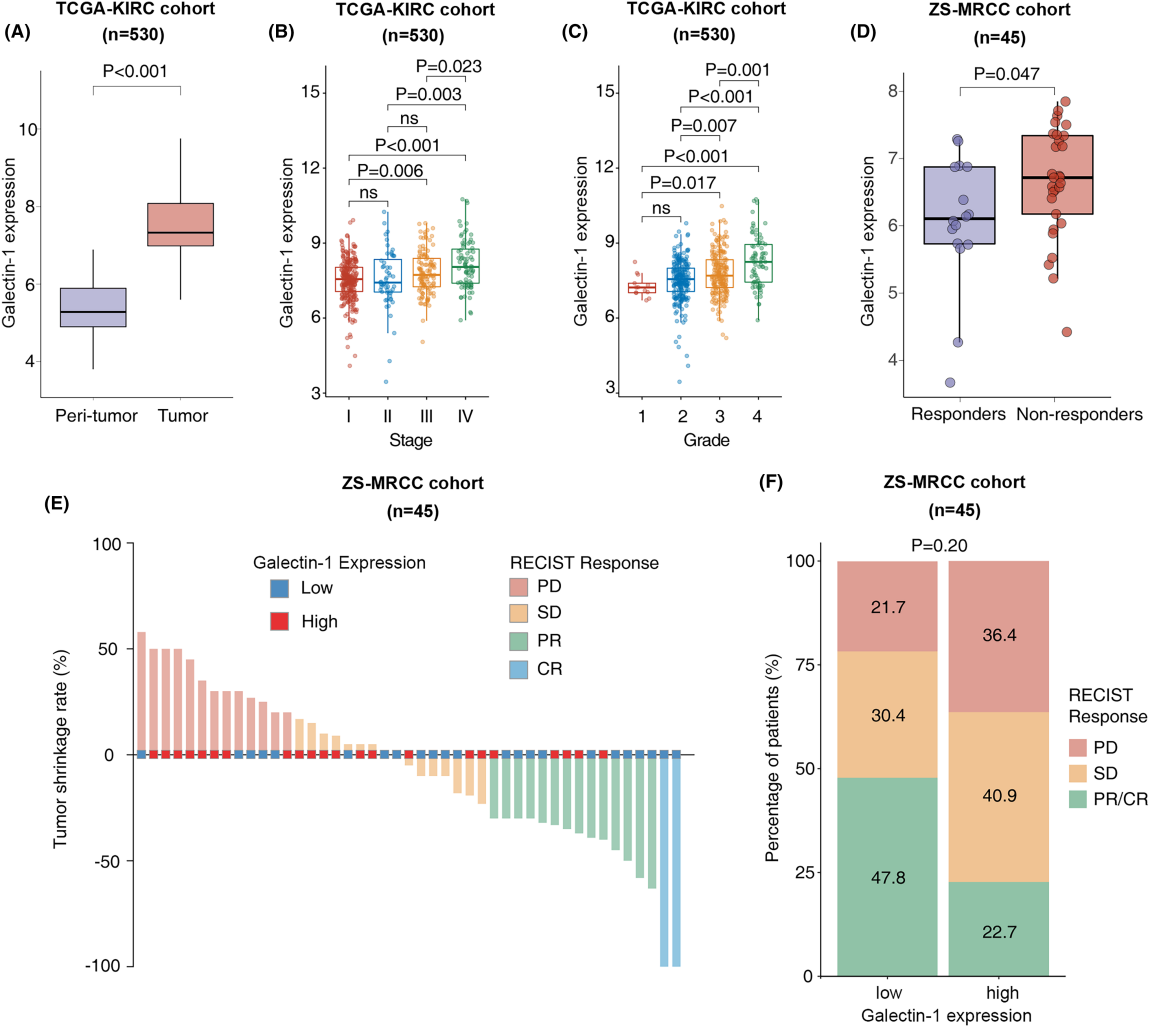
Galectin‐1 expression associated with progression and resistance of IO/TKI therapy in RCC. (A) Expression of galectin‐1 in tumor tissues and adjacent non‐tumor tissues of RCC in the TCGA‐KIRC cohort. *p*‐value, Wilcoxon signed‐rank test. (B) Association between galectin‐1 expression and tumor stage in the TCGA‐KIRC cohort. *p*‐values, Kruskal–Wallis *H*‐test. (C) Association between galectin‐1 expression and tumor grade in the TCGA‐KIRC cohort. *p*‐values, Kruskal–Wallis *H*‐test. (D) Expression of galectin‐1 in different responders and non‐responders of IO/TKI therapy in the ZS‐MRCC cohort. *p*‐value, Wilcoxon signed‐rank test. (E) Galectin‐1 expression, tumor shrinkage rate and RECIST response of IO/TKI therapy in the ZS‐MRCC cohort. (F) Heterogenous RECIST response of IO/TKI therapy between the high‐ and low‐galectin‐1 groups in the ZS‐MRCC cohort. *p*‐values, Kruskal–Wallis *H*‐test.

Expression of galectin‐1 was significantly elevated in RCC tissues, compared with peri‐tumor tissues, in the TCGA‐KIRC cohort (*p* < 0.001, Figure 1A). Moreover, galectin‐1 was correlated with advanced disease in the TCGA‐KIRC cohort (Figure 1B). In stage IV disease, expression of galectin‐1 was higher than stage I (*p* < 0.001), stage II (*p* = 0.003) or stage III (*p* = 0.023) disease (Figure 1B). Furthermore, higher galectin‐1 expression was also correlated with more aggressive disease in the TCGA‐KIRC cohort (Figure 1C). In grade 3 RCC, galectin‐1 expression was higher than grade 1 (*p* = 0.017) or grade 2 (*p* = 0.007) (Figure 1C). Similarly, in grade 4 RCC, galectin‐1 expression was also higher than grade I (*p* < 0.001), grade II (*p* < 0.001) or grade III (*p* = 0.001) (Figure 1C).

### 3.2 Elevated galectin‐1 expression correlated with resistance of IO/TKI therapy

IO/TKI therapy only shows response in part of patients, with no clinical available predictive biomarker. Galectin‐1 regulates antitumor immune response in human neoplasms. In the ZS‐MRCC cohort, galectin‐1 expression was elevated in non‐responders of IO/TKI therapy (non‐responders vs. responders, *p* = 0.047; Figure 1D). After classification into high and low galectin‐1 groups, according to median value, low galectin‐1 tumors were more likely to show remission in the ZS‐MRCC cohort (Figure 1E). According to RECIST 1.1 criteria, therapeutic response was classified as complete response (CR), partial response (PR), stable disease (SD), or progressive disease (PD) (Figure 1E). Compared with high galectin‐1 group, low galectin‐1 group showed trend of CR/PR (47.8% vs. 22.7%), although not statistically significant (Figure 1F) in the ZS‐MRCC cohort.

### 3.3 Elevated galectin‐1 indicated poor survival of IO/TKI therapy

**FIGURE 2:**
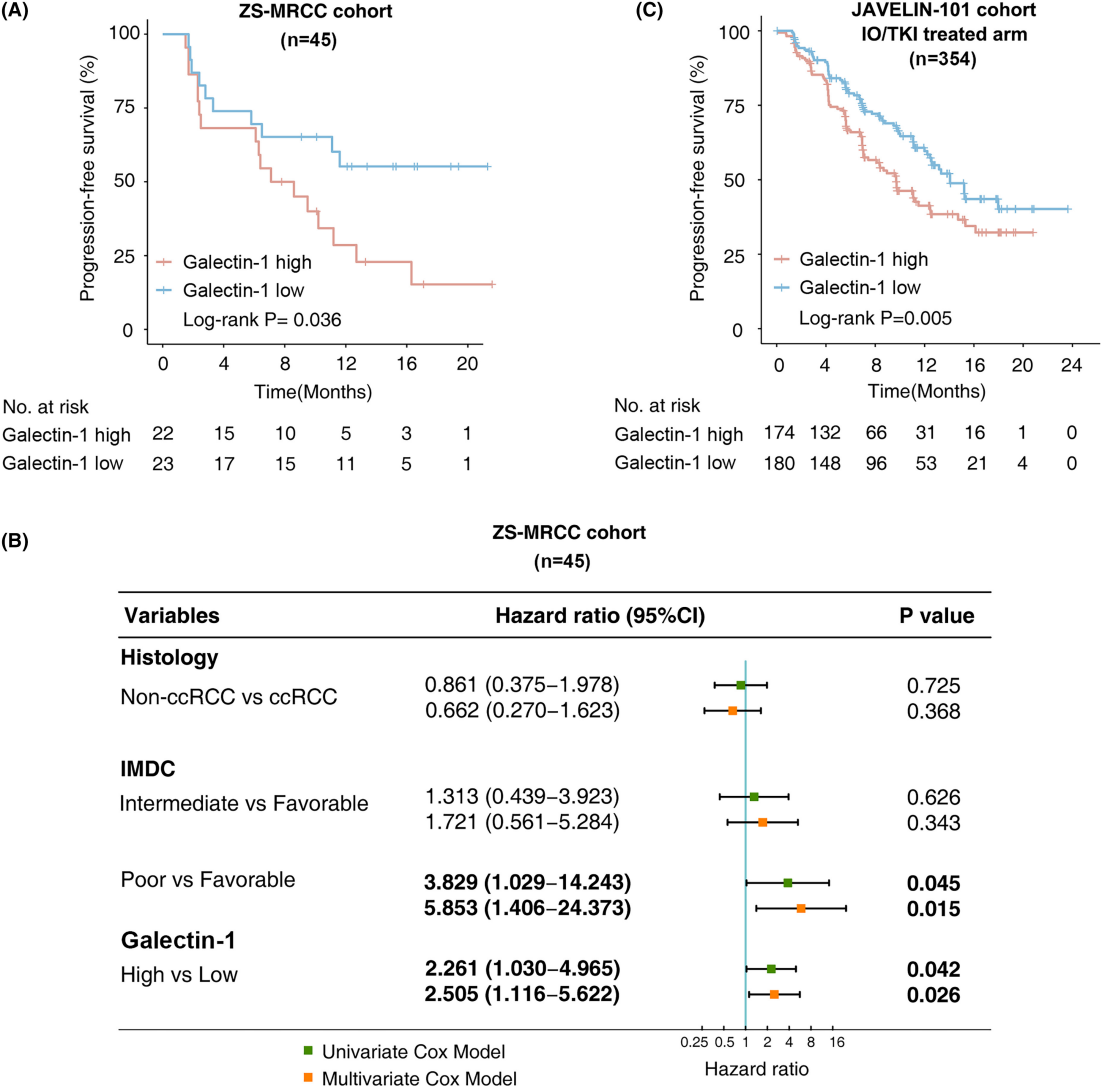
Prognostic relevance of galectin‐1 expression for IO/TKI therapy in RCC. (A) Progression‐free survival according to galectin‐1 expression level in the ZS‐MRCC cohort. *p*‐value, log‐rank test. (B) Univariate and multivariate survival analysis for RCC treated by IO/TKI therapy in the ZS‐MRCC cohort. ccRCC, clear cell renal cell carcinoma. Hazard ratio and *p*‐values, Cox regression analysis. (C) Progression‐free survival according to galectin‐1 expression in the IO/TKI treated arm of the JAVELIN‐101 cohort. *p*‐value, log‐rank test.

In our ZS‐MRCC cohort, high galectin‐1 indicated poor PFS (log‐rank *p* = 0.036, Figure 2A), and higher risk of progression (Cox regression hazard ratio (HR) 2.261, 95% confidence interval (CI) 1.030–4.965, *p* = 0.042, Figure 2B). To exclude bias from other prognostic factors, such as tumor histology (clear cell renal cell carcinoma (ccRCC) or non‐clear cell renal cell carcinoma (non‐ccRCC)) and International Metastatic RCC Database Consortium (IMDC) risk group, multivariate Cox regression analysis was performed. Galectin‐1 was demonstrated as an independent prognostic factor for IO/TKI therapy (HR 2.505, 95% CI 1.116–5.622, *p* = 0.026, Figure 2B). In the validation JAVELIN‐101 cohort, high galectin‐1 also indicated shorter PFS (log‐rank *p* = 0.005, Figure 2C). According to these results, elevated galectin‐1 could indicated poor survival of IO/TKI therapy.

### 3.4 Galectin‐1 was not correlated with T cell infiltration

**FIGURE 3:**
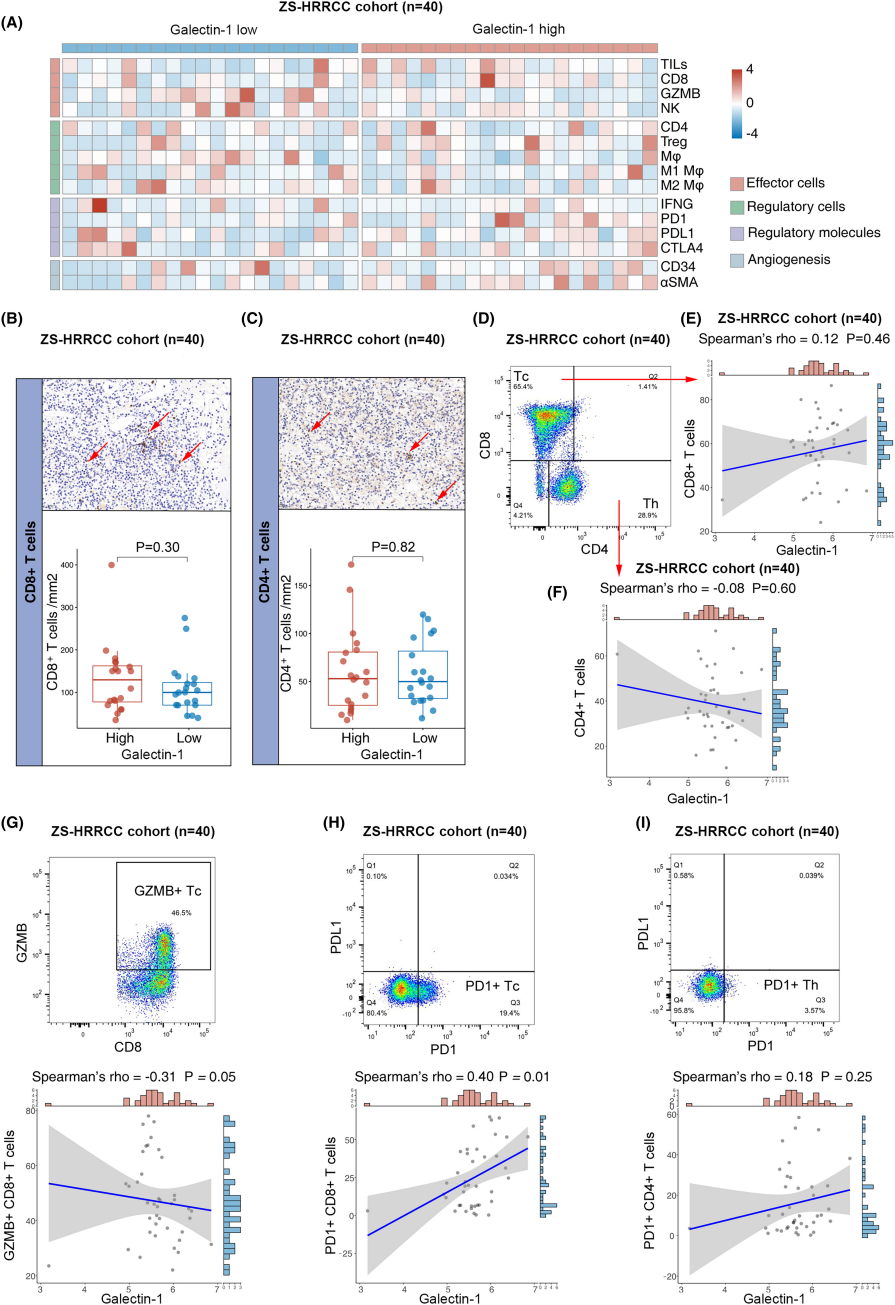
Galectin‐1 expression correlated with CD8+ T cell exhaustion and dysfunction in RCC. (A) Immune cell infiltration and immunoregulatory factors in RCC according to galectin‐1 expression in the ZS‐HRRCC cohort. (B, C) Quantification of CD8^+^ T cells (B) and CD4^+^ T cells (C) by immunohistochemistry, and association with galectin‐1 expression in the ZS‐HRRCC cohort. *p*‐values, Wilcoxon signed‐rank test. (D) Flow cytometric gating strategy of CD8^+^ T cells and CD4^+^ T cells by flow cytometry in the ZS‐HRRCC cohort. (E, F) Correlation between CD8^+^ T cells (E), CD4^+^ T cells (F) and galectin‐1 expression in the ZS‐HRRCC cohort. *p*‐values and *ρ*, Spearman's correlation test. (G, I) Flow cytometric gating strategy and correlation between GZMB^+^CD8^+^ T cells (G), PD1^+^CD8^+^ T cells (H), PD1^+^CD4^+^ cells (I) and galectin‐1 expression in the ZS‐HRRCC cohort. *p*‐values and *ρ*, Spearman's correlation test.

The effect of immunotherapy highly relies on TME status. The relationship between galectin‐1 and TME components, such as effector cells, regulatory cells, regulatory molecules and angiogenesis, was estimated by IHC in the ZS‐HRRCC cohort (Figure 3A). By IHC, neither CD8+ T cells (*p* = 0.30, Figure 3B) nor CD4+ T cells (*p* = 0.82, Figure 3C) showed significant correlation with galectin‐1. Flow cytometry was further performed to determine the infiltration of CD8+ and CD4+ T cells (Figure 3D). Similar with results of IHC, neither CD8+ T cells (Spearman's *ρ* = 0.12, *p* = 0.46, Figure 3E) nor CD4+ T cells (Spearman's *ρ* = −0.08, *p* = 0.60, Figure 3F) showed significant correlation with galectin‐1.

### 3.5 Galectin‐1 correlated with dysfunctional CD8+ T cells

The functional status of tumor‐infiltrating lymphocytes was further determined by flow cytometry in the ZS‐HRRCC cohort. The number of cytotoxic GZMB+CD8+ T cells was found negatively correlated with galectin‐1 expression (Spearman's *ρ* = −0.31, *p* = 0.05, Figure 3G). Moreover, the number of PD1 + CD8+ T cells was found positively correlated with galectin‐1 expression (Spearman's *ρ* = 0.40, *p* = 0.01, Figure 3H). The results indicated the correlation between galectin‐1 and dysfunctional CD8+ T cells. On the other hand, the number of PD1 + CD4+ T cells did not correlate with galectin‐1 expression (Spearman's *ρ* = 0.18, *p* = 0.25, Figure 3I).

### 3.6 Suppressive TME in RCC with elevated galectin‐1 expression

**FIGURE 4:**
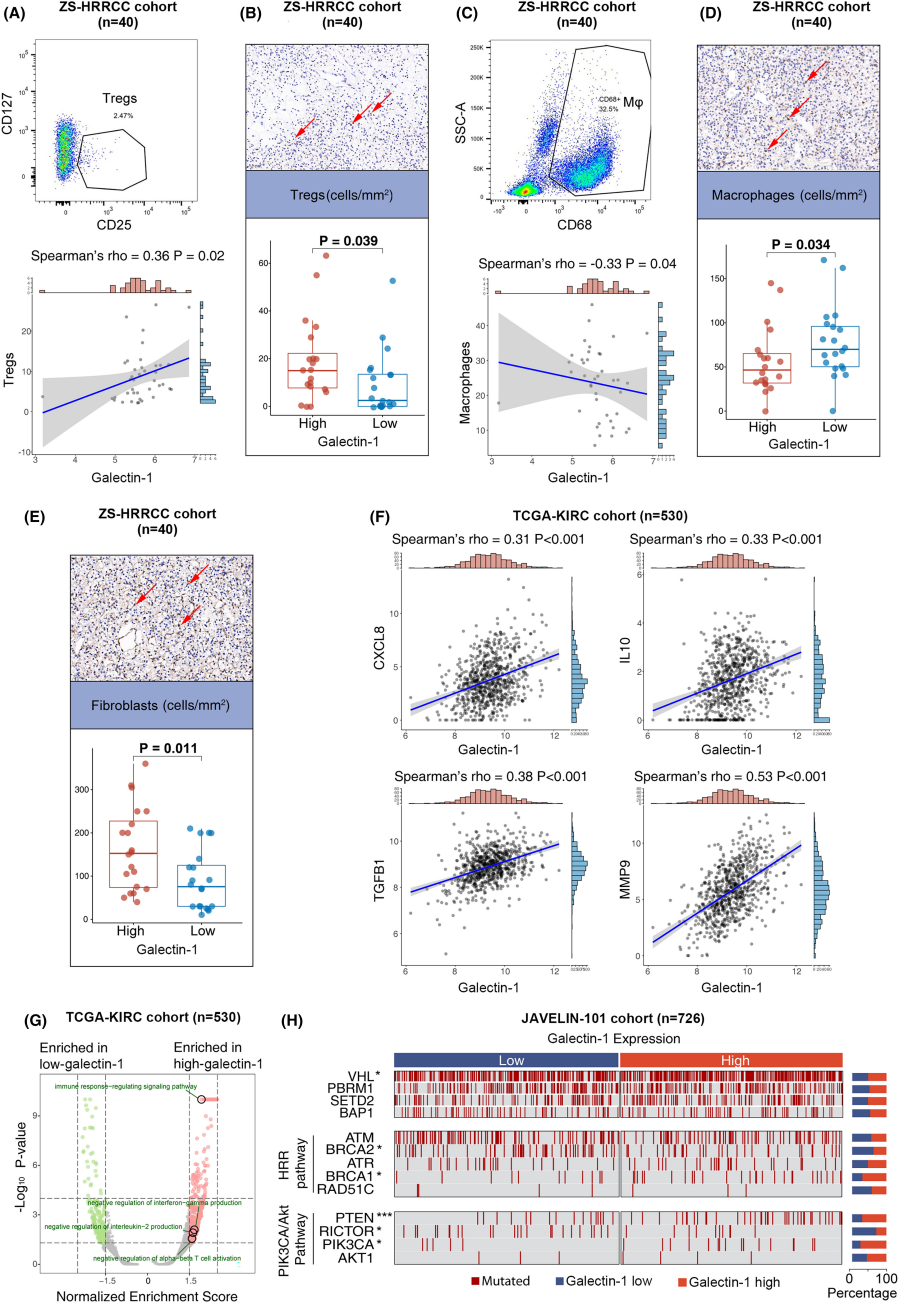
Suppressive tumor microenvironment in RCC with elevated galectin‐1 expression. (A) Flow cytometric gating strategy of regulatory T cells and correlation with galectin‐1 expression in the ZS‐HRRCC cohort. *p*‐value and *ρ*, Spearman's correlation test. (B) Immunohistochemical quantification of regulatory T cells and association with galectin‐1 expression in the ZS‐HRRCC cohort. *p*‐value, Wilcoxon signed‐rank test. (C) Flow cytometric gating strategy of macrophages, and correlation with galectin‐1 expression in the ZS‐HRRCC cohort. *p*‐value and *ρ*, Spearman's correlation test. (D) Immunohistochemical quantification of macrophages and association with galectin‐1 expression in the ZS‐HRRCC cohort. *p*‐value, Wilcoxon signed‐rank test. (E) Immunohistochemical quantification of α‐SMA+ fibroblasts and association with galectin‐1 expression in the ZS‐HRRCC cohort. *p*‐value, Wilcoxon signed‐rank test. (F) Correlation between CXCL8, IL10, TGFB1, MMP9, and galectin‐1 expression in the TCGA‐KIRC cohort. *p*‐values and *ρ*, Spearman's correlation test. (G) Gene‐set enrichment analysis of high galectin‐1 versus low galectin‐1 samples in the TCGA‐KIRC cohort. Red plots represent pathways enriched in high galectin‐1 samples, and green plots represent pathways enriched in low galectin‐1 samples. (H) Somatic mutations sorted according to galectin‐1 expression in the JAVELIN‐101 cohort. *p*‐values, Chi‐square test. *, *p* < 0.05; ***, *p* < 0.001.

Suppressive TME leads to dysfunctional T cells. In the study, regulatory cells in TME were also evaluated by flow cytometry and IHC in the ZS‐HRRCC cohort. The infiltration of regulatory T cells was elevated in samples with high galectin‐1 expression, by flow cytometry (Spearman's *ρ* = 0.36, *p* = 0.02, Figure 4A) and IHC (*p* = 0.039, Figure 4B). However, the infiltration of macrophages was decreased in high galectin‐1 samples, by flow cytometry (Spearman's *ρ* = −0.33, *p* = 0.04, Figure 4C) and IHC (*p* = 0.034, Figure 4D). Interestingly, the number of fibroblasts was also increased in high galectin‐1 samples (*p* = 0.011, Figure 4E).

The correlation between galectin‐1 and suppressive molecules was also evaluated in RCC.

Positive correlation was found between galectin‐1 and CXCL8 (Spearman's *ρ* = 0.31, *p* < 0.001) and IL10 (Spearman's *ρ* = 0.33, *p* < 0.001) in the TCGA‐KIRC cohort (Figure 4F). Moreover, galectin‐1 was positively correlated with transforming growth factor family gene TGFB1 (Spearman's *ρ* = 0.38, *p* < 0.001), as well as extracellular matrix remodeling gene MMP9 (Spearman's *ρ* = 0.53, *p* < 0.001) (Figure 4F).

### 3.7 Pathway enrichment and genomic mutations in RCC with elevated galectin‐1 expression

To define enriched pathways in high galectin‐1 tumors, GSEA was further performed. Several immune‐regulating pathways were found enriched in high galectin‐1 samples in the TCGA‐KIRC cohort, such as negative regulation of alpha‐beta T cell activation, negative regulation of interleukin‐2 production, negative regulation of interferon‐gamma production, and immune response‐regulating signaling pathway (Figure 4G).

Correlation between genomic mutations and galectin‐1 expression status in the JAVELIN‐101 cohort is illustrated in Figure 4H. Mutation of VHL was more prevalent in high galectin‐1 samples (*p* < 0.05, Figure 4H). Interestingly, among genes of homologous recombinational repair pathway, BRCA1 was more prevalent in high galectin‐1 samples (*p* < 0.05, Figure 4H), but BRCA2 was more prevalent in low galectin‐1 samples (*p* < 0.05, Figure 4H). Among genes of PIK3CA/AKT pathway, higher rate of PTEN (*p* < 0.001) and PIK3CA mutation (*p* < 0.05), and lower rate of RICTOR mutation (*p* < 0.05) were found in high galectin‐1 samples.

### 3.8 Integrated risk model for RCC treatment selection

**FIGURE 5:**
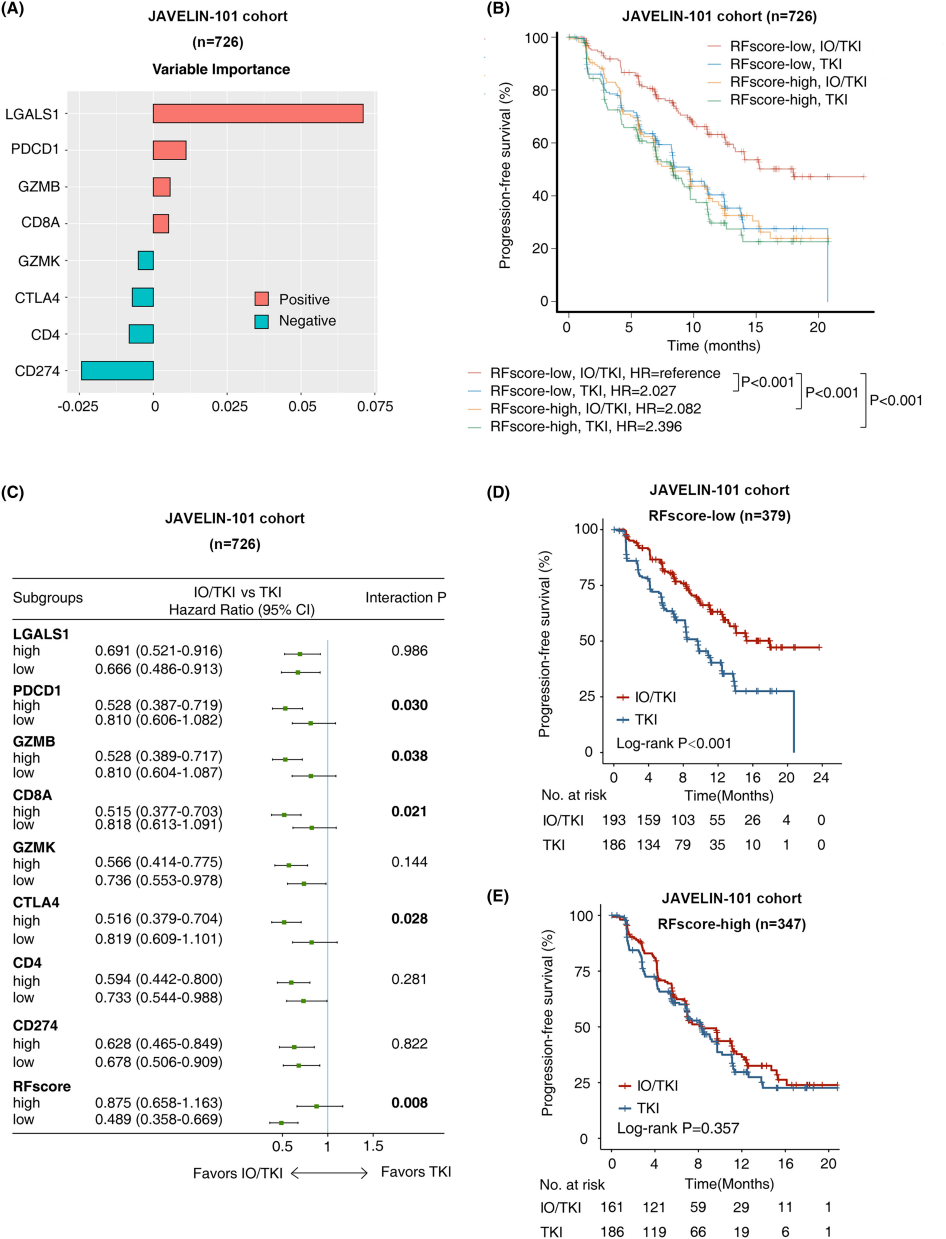
An integrated risk model for IO/TKI benefit versus TKI monotherapy. (A) Construction and variables' importance of the random forest score (RFscore) involving LGALS1, PDCD1, GZMB, CD8A, GZMK, CTLA4, CD4, and CD274 expression in the JAVELIN‐101 cohort. (B) Progression‐free survival according to high‐ and low‐RFscore in patients treated by IO/TKI in the JAVELIN‐101 cohort. The red line represents patients with RFscore‐low and treated by IO/TKI, the blue line represents patients with RFscore‐low and treated by TKI, the yellow line represents patients with RFscore‐high and treated by IO/TKI, and the green line represents patients with RFscore‐high and treated by TKI. *p*‐value, log‐rank test. (C) Benefit of IO/TKI versus TKI monotherapy, according to prognostic factors and the integrated RFscore in the JAVELIN‐101 cohort. Hazard ratio and interaction P values, Cox regression. (D, E) Progression‐free survival of IO/TKI compared with TKI monotherapy in the low‐RFscore group (D) and the high‐RFscore group (E) in the JAVELIN‐101 cohort. *p*‐values, log‐rank test.

Treatment selection based on molecular subtypes could lead to survival benefit, in RCC treated by IO monotherapy, or TKI monotherapy.^26^ When it comes to IO/TKI combination therapy, no predictive biomarker is available. We applied random forest algorithm in the JAVELIN‐101 cohort, in order to build a novel risk model for treatment selection between IO/TKI and TKI monotherapy. The random forest model (RFscore) integrated expression of genes including LGALS1, PDCD1, GZMB, CD8A, GZMK, CTLA4, CD4, and CD274 (Figure 5A). Patients with low‐RFscore and treated by IO/TKI showed the optimal prognosis in the JAVELIN‐101 cohort, compared with other patients (*p* < 0.001, Figure 5B).

Both IO/TKI and TKI monotherapy are first‐line recommendations for metastatic RCC. However, no parameter has been applied for treatment selection. Among the genes of RFscore, PDCD1 (interaction *p* = 0.030), GZMB (interaction *p* = 0.038), CD8A (interaction *p* = 0.021) and CTLA4 (interaction *p* = 0.028) showed predictive value for IO/TKI benefit versus TKI monotherapy (Figure 5C). After integration, in the low‐RFscore group, IO/TKI exceeded TKI monotherapy for PFS in the JAVELIN‐101 cohort (HR = 0.489, 95%CI 0.358–0.669, log‐rank *p* < 0.001, Figure 5C,D). However, in the high‐RFscore group, IO/TKI did not exceed TKI in the JAVELIN‐101 cohort (HR = 0.875, 95%CI 0.658–1.163, log‐rank *p* = 0.357, Figure 5C,E). In conclusion, the integrated RFscore may be used as a potential predictive for IO/TKI benefit in RCC.

## 4 DISCUSSION

Galectin‐1, encoded by LGALS1 gene, is a critical immunosuppressive molecule across cancer types.^9^ Its prognostic and predictive role in RCC treated with IO/TKI has not yet been determined. In the study, galectin‐1 was discovered to be prognostic in two independent mRCC cohorts treated with IO/TKI combinations. Galectin‐1 was also linked to IO/TKI therapy resistance. In addition, galectin‐1 expression was related with CD8+ T cell dysfunction and immune evasion in the RCC microenvironment. Furthermore, the RFscore developed using galectin‐1 and other immunologic characteristics demonstrated predictive significance for therapeutic advantages of IO/TKI versus TKI monotherapy in RCC.

Although TKIs have traditionally been the preferred as first‐line therapy, the recent clinical trials such KEYNOTE 426,^6^ JAVELIN 101,^5^ CheckMate 9ER,^27^ and CLEAR^28^ have successfully established the role of IO/TKI combos as first‐line therapy in advanced RCC. However, a significant proportion of patients continued to fail to respond to IO/TKI combinations (PD 28.9% and SD 35.6%), highlighting the critical need for prognostic and predictive biomarkers of IO/TKI combination therapy. As an immunosuppressive molecule, galectin‐1 was also associated with response of IO monotherapy.^11^, ^13^ However, the prognostic and predictive effect of galectin‐1 for IO/TKI combinations in RCC has not yet been investigated. In the current study, galectin‐1 was found associated with poor PFS in two independent cohorts of advanced RCC. Moreover, the integrated RFscore based on galectin‐1 expression and immunogenic genes predicted benefit of IO/TKI versus TKI monotherapy. These findings identified galectin‐1 expression as a possible prognostic and predictive factor for IO/TKI therapy in RCC. However, the findings should be verified in prospective, large‐scale studies.

Galectin‐1, encoded by the LGALS1 gene, is an important immunosuppressive molecule across cancer types.^9^ Tumor‐secreted galectin‐1 can impair immune cell function in the tumor microenvironment.^10^, ^11^ Furthermore, galectin‐1 has been shown to cause T cell apoptosis and impede T cell receptor (TCR) signal transmission.^29^ In the current study, we also found that galectin‐1 was associated with exhaustion and reduced function of CD8^+^ T cells, manifested as high PD1 expression and low GZMB expression. These results also demonstrated the immunosuppressive role of galecin‐1 in RCC. However, no correlation was found between galectin‐1 expression and CD4^+^ T cell exhaustion. The varied effects of galectin‐1 on CD8^+^ and CD4^+^ T cells could be attributed to distinct glycoprotein receptors on these cells, which need to be explored further.

Previous studies have shown that galectin‐1 is abundantly expressed in tumor cells as well as in cancer‐associated fibroblasts (CAFs).^30^, ^31^ Galectin‐1 produced by cancer cells could activate CAFs and induce the tryptophan 2,3‐dioxygenase/kynurenine axis, which inhibited T cell differentiation and function, contributing to immunosuppression.^32^ In addition, galectin‐1 treatment induced the expansion of Tregs and secretion of Th2 cytokines.^12^ The current study also discovered the correlation between galectin‐1 expression and CAFs as well as Tregs. Galectin‐1 expression was also found associated with immune‐suppressive cytokines including CXCL8, IL10 and TGFB1, as well as the matrix remodeling enzyme MMP9. The secretion of galectin‐1 and activation of downstream pathways contributes to matrix remodeling and T cell exclusion.^11^ Nonetheless, exact mechanisms should be clarified in future mechanistic investigations.

Galectin‐1 mainly shows immunosuppressive roles in the tumor microenvironment^9^ and was regarded as a promising immune checkpoint target to improve T‐cell treatment.^33^ Galectin‐1 has been investigated as a possible target for synergistic chemo‐immunotherapy in hepatocellular carcinoma.^34^ However, the recent research has mainly focused on the expression and effect of galectin‐1 in pre‐treatment samples. The dynamic changes of galectin‐1 expression and its dynamic roles throughout immunotherapy are still unknown. Given that immune checkpoint inhibitors can reactivate antitumor immunity in RCC, we anticipate galectin‐1's role may shift throughout immunotherapy. However, our study only assessed the expression of galectin‐1 in pre‐treatment RCC samples. We would like to evaluate the dynamic changes of galectin‐1 expression and its dynamic roles during immunotherapy in our further studies.

The major limitation of the study is the retrospective design, which may lead to enrollment and recall biases. However, all the patients in the ZS‐MRCC cohort were from the same institution, and all the patients received consistent follow‐up procedures. These approaches may reduce the potential of recall bias. Moreover, the prognostic role of galectin‐1 was further confirmed in the JAVELIN‐101 validation cohort, which was derived from a prospective, phase III randomized control trial. These efforts may reduce the potential of biases. Secondly, as the EAU guideline of RCC recommended IO/TKI combinations as first‐line therapy in mRCC just recently, the study could only collect IO/TKI treated patients in a short period of time, leading to the limited sample size. Further prospective validation studies in larger cohorts are still required. Besides, the RFscore showed predictive value for survival benefit between combined IO/TKI therapy and TKI monotherapy in the JAVELIN‐101 cohort, which should also be validated in further prospective studies. Moreover, the study investigated the correlation between galectin‐1 expression and tumor microenvironment in the ZS‐HRRCC cohort by IHC and flow cytometry, but these preliminary findings and the underlying mechanisms should be validated in future studies. In addition, to improve the feasibility of galecitn‐1 analysis, we are also planning to evaluate the application of IHC staining for galectin‐1 expression in the clinic. The study also showed the probability of the combination of anti‐galectin‐1 treatment with IO/TKI, which has no clinical application yet. We expect to investigate it in future studies.

## 5 CONCLUSIONS

High galectin‐1 expression suggested treatment resistance and a shorter PFS for IO/TKI therapy in mRCC. High galectin‐1 expression was also linked to CD8+ T cell dysfunction and immune evasion in RCC patients. The integrated RFscore of galectin‐1 expression and other immunologic variables may influence treatment decision between IO/TKI and TKI monotherapy in mRCC.

## AUTHOR CONTRIBUTIONS

**Jiajun Wang:** Conceptualization (equal); data curation (equal); formal analysis (equal); funding acquisition (equal); investigation (equal); methodology (equal); project administration (equal); resources (equal); visualization (equal); writing – original draft (equal); writing – review and editing (equal). **Sihong Zhang:** Data curation (equal); formal analysis (equal); investigation (equal); resources (equal). **Ying Wang:** Conceptualization (equal); data curation (equal); formal analysis (equal); funding acquisition (equal); investigation (equal); methodology (equal); resources (equal). **Yanjun Zhu:** Methodology (equal); project administration (equal); resources (equal); writing – review and editing (equal). **Xianglai Xu:** Conceptualization (equal); data curation (equal); funding acquisition (equal); investigation (equal); methodology (equal); resources (equal); software (equal); validation (equal). **Jianming Guo:** Funding acquisition (equal); project administration (equal); resources (equal); supervision (equal); validation (equal).

## FUNDING INFORMATION

This study was funded by grants from National Natural Science Foundation of China (81902898, 82200090, 81700660, 81772696, 81974393, 82272776), Shanghai Municipal Health Commission (2020CXJQ03), Shanghai Sailing Program (19YF1407900) and China Urological Oncology Research Fund (H2023‐018). All the sponsors have no roles in the study design, in the collection, analysis, or in the interpretation of data.

## CONFLICT OF INTEREST STATEMENT

The authors declare no potential conflict of interest.

## ETHICS STATEMENT

The study followed the Declaration of Helsinki and was approved by the Clinical Research Ethics Committee of Zhongshan Hospital, Fudan University (B2021‐119). Informed consent was obtained from each participate.

## IMPACT STATEMENT

High galectin‐1 expression indicated therapeutic resistance and shorter progression‐free survival of IO/TKI therapy in renal cell carcinoma. High galectin‐1 also indicated CD8+ T cell dysfunction, and infiltration of regulatory T cells and fibroblasts. High galectin‐1 could be applied for patient selection of IO/TKI therapy in RCC.

## Supporting information


Table S1.

Table S2.

## Data Availability

Data of the study can be shared to other researches upon reasonable request, according to data sharing policy.
