# Supplementary material for: Effect of galectin‐1 on prognosis and responsiveness of immune checkpoint plus tyrosine kinase inhibition in renal cell carcinoma
Source: Cancer Med. 2024 Mar 28;13(7):e7113. doi: 10.1002/cam4.7113 (PMC10974699; doi:10.1002/cam4.7113)
Supplement: Supplementary file 2 — Table S2. [file CAM4-13-e7113-s002.doc]

| Supplementary Table S2. Information of antibodies. | | | | | |
| --- | --- | --- | --- | --- | --- |
| Name | Company | Catalogue No. | Application | Diluted |  |
| Fixable Viability Dye, eFluor780 | ThermoFisher Scientific | 65-0865-18 | FC | 1:200 |  |
| Mouse anti-human CD45, BV510 | BioLegend | 368526 | FC† | 1:100 |  |
| Mouse anti-human CD68, AF647 | BioLegend | 333820 | FC | 1:100 |  |
| Mouse anti-human CD3, BUV395 | BD Biosciences | 564001 | FC | 1:100 |  |
| Mouse anti-human CD4, AF700 | ThermoFisher Scientific | 56-0049-42 | FC | 1:100 |  |
| Mouse anti-human CD8, PerCP-Cy5.5 | ThermoFisher Scientific | 45-0088-42 | FC | 1:100 |  |
| Mouse anti-human CD279, BV421 | BD Biosciences | 564323 | FC | 1:100 |  |
| Mouse anti-human CD274, PE | ThermoFisher Scientific | 12-5983-42 | FC | 1:100 |  |
| Mouse anti-human CD25, PE-Cy7 | ThermoFisher Scientific | 25-0259-42 | FC | 1:100 |  |
| Mouse anti-human CD127, BV711 | BD Biosciences | 563165 | FC | 1:100 |  |
| Mouse anti-human/mouse Granzyme B, FITC | BioLegend | 515403 | FC | 1:200 |  |
| Rabbit anti-human Granzyme | Abcam | ab208586 | IHC* | 1:250 |  |
| Rabbit anti-human/mouse Baf180 | Abcam | ab243876 | IHC | 1:200 |  |
| Rabbit anti-human/mouse/rat CD86 | Bioss antibodies | bs-1035R | IHC | 1:200 |  |
| Rabbit anti-human CD163 | Servicebio | GB113152 | IHC | 1:500 |  |
| Rabbit anti-human IFNγ | Bioss antibodies | bs-0388R | IHC | 1:200 |  |
| Rabbit anti-human/mouse CTLA4 | Bioss antibodies | bs-1179R | IHC | 1:200 |  |
| Rabbit anti-human/mouse/rat Ki67 | Servicebio | GB111499 | IHC | 1:300 |  |
| Rabbit anti-human CD8 | Servicebio | GB13068 | IHC | 1:100 |  |
| Rabbit anti-human CD68 | Servicebio | GB113150 | IHC | 1:500 |  |
| Mouse anti-human FOXP3 | Abcam | ab20034 | IHC | 1:100 |  |
| Mouse anti-human CD56 | Servicebio | GB12041 | IHC | 1:600 |  |
| Mouse anti-human PD1 | Abcam | ab52587 | IHC | 1:50 |  |
| Mouse anti-human PDL1 | Servicebio | GB14132 | IHC | 1:200 |  |
| Mouse anti-human CD4 | Servicebio | GB13064-1 | IHC | 1:100 |  |
| Mouse anti-human CD34 | Servicebio | GB121693 | IHC | 1:500 |  |
| Rabbit anti-humanα-SMA | Servicebio | GB111364 | IHC | 1:500 |  |
| †FC, flow cytometry. *IHC, immunohistochemistry. | | | | | |
